# LINE-1 Methylation Status Correlates Significantly to Post-Therapeutic Recurrence in Stage III Colon Cancer Patients Receiving FOLFOX-4 Adjuvant Chemotherapy

**DOI:** 10.1371/journal.pone.0123973

**Published:** 2015-04-28

**Authors:** Yun-Ting Lou, Chao-Wen Chen, Yun-Ching Fan, Wei-Chiao Chang, Chien-Yu Lu, I-Chen Wu, Wen-Hung Hsu, Ching-Wen Huang, Jaw-Yuan Wang

**Affiliations:** 1 Graduate Institute of Genomic Medicine, College of Medicine, Kaohsiung Medical University, Kaohsiung, Taiwan; 2 Department of Ophthalmology, E-DA Hospital, I-Shou University, Kaohsiung, Taiwan; 3 Department of Surgery, Kaohsiung Medical University Hospital, Kaohsiung Medical University, Kaohsiung, Taiwan; 4 Department of Surgery, Faculty of Medicine, College of Medicine, Kaohsiung Medical University, Kaohsiung, Taiwan; 5 Department of Emergency Medicine, Faculty of Medicine, College of Medicine, Kaohsiung Medical University, Kaohsiung, Taiwan; 6 Department of Clinical Pharmacy, School of Pharmacy, Taipei Medical University, Taipei, Taiwan; 7 Department of Pharmacy, Taipei Medical University-Wanfang Hospital, Taipei, Taiwan; 8 Cancer Center, Kaohsiung Medical University Hospital, Kaohsiung Medical University, Kaohsiung, Taiwan; 9 Master Program for Clinical Pharmacogenomics and Pharmacoproteomics, School of Pharmacy, Taipei Medical University, Taipei, Taiwan; 10 Department of Internal Medicine, Faculty of Medicine, College of Medicine, Kaohsiung Medical University, Kaohsiung, Taiwan; 11 Division of Gastroenterology, Department of Internal Medicine, Kaohsiung Medical University Hospital, Kaohsiung Medical University, Kaohsiung, Taiwan; 12 Division of Gastrointestinal and General Surgery, Department of Surgery, Kaohsiung Medical University Hospital, Kaohsiung Medical University, Kaohsiung, Taiwan; 13 Graduate Institute of Medicine, College of Medicine, Kaohsiung Medical University, Kaohsiung, Taiwan; 14 Graduate Institute of Clinical Medicine, College of Medicine, Kaohsiung Medical University, Kaohsiung, Taiwan; 15 Center for Biomarkers and Biotech Drugs, Kaohsiung Medical University, Kaohsiung, Taiwan; University of Crete, GREECE

## Abstract

**Background:**

Methylation levels of long interspersed nucleotide elements (LINE-1) are representative of genome-wide methylation status and crucial in maintaining genomic stability and expression. Their prognostic impact on colon cancer patients receiving adjuvant chemotherapy has not been well established. We evaluated the association between LINE-1 methylation status and clinicopathologic features and postoperative oncological outcomes in stage III colon cancer patients.

**Materials and Methods:**

129 UICC stage III colon cancer patients who had received radical resection and FOLFOX adjuvant chemotherapy were enrolled. Global methylation was estimated by analyzing tumor LINE-1 methylation status using bisulfite-polymerase chain reaction (PCR) and pyrosequencing assay. Demographics, clinicopathological data, and postoperative outcomes were recorded by trained abstractors. Outcome measurements included postoperative recurrence and disease-free survival. Univariate, multivariate, and survival analyses were conducted to identify prognostic factors of oncological outcomes.

**Results:**

The LINE-1 methylation of all 129 patients was measured on a 0–100 scale (mean 63.3; median 63.7, standard deviation 7.1), LINE-1 hypomethylation was more common in patients aged 65 years and above (61.7%±7.6% vs. 64.6±6.4, *p*=0.019) and those with post-therapeutic recurrence (61.7±7.4 vs 64.3±6.7, *p*=0.041). Considering risk adjustment, LINE-1 hypomethylation was found to be an independent risk factor of post-therapeutic recurrence (Adjusted OR=14.1, *p*=0.012). Kaplan-Meier analysis indicated that patients in the low methylation group had shorter period of disease free survival (*p*=0.01). In a stratified analysis that included 48 patients with post-therapeutic recurrence, it was found that those who experienced shorter period of disease free survival (≦6 months) appeared to have lower LINE-1 methylation levels than patients who reported of recurrence after 6 months (56.68±15.75 vs. 63.55±7.57, *p*=0.041)

**Conclusion:**

There was a significantly greater risk of early postoperative recurrence and a shorter period of disease-free survival in Stage III colon cancer patients exhibiting LINE-1 hypomethylation status after being treated with radical resection and FOLFOX chemotherapy.

## Introduction

There has been a steady rise in colorectal cancer (CRC) cases worldwide in recent years. According to GLOBOCAN estimates presented in 2008, CRC is the third most common cancer in males and the second most common in females [1]. Owing to the proliferation of unhealthy diets and lifestyles in Taiwan, CRC has become the third most common cause of cancer-related deaths. In recent years, more than 4,000 CRC patients have died annually (http://www.doh.gov.tw/statistic/index.htm; accessed in December 2013). Recent improvements in tumor detection and adjuvant therapy, however, have increased the life expectancies of CRC patients [2]. However, despite curative surgery and adjuvant chemotherapy, there are still a considerable number of patients who encounter tumor recurrence during their follow-up period. Many postoperative surveillance programs have been proposed for detecting the possibility of recurrence at an early stage. Medical researchers have also reached a consensus on routine surveillance of stage II and III CRC patients who have been treated with curative surgery [3–5]. At the same time, many researchers continue to search for sophisticated biomarkers capable of effectively detecting asymptomatic recurrence of CRC after surgery. Presently, some biomarkers are promising but still far from being assessable in most patients. Meanwhile, the labeling of high-risk groups which are prone to relapse appears to be another tactic among the intensive postoperative surveillance strategies used in these susceptible populations.

CRC develops due to genetic and epigenetic alterations. In the last decade, many studies have investigated the role of epigenetic factors in the development of CRC in patients [6,7]. Epigenetics, which has been described as a bridge between genetics and the environment, has been found to have a close relation with cancer biology [8,9]. Epigenetic alterations could be inherited but these factors are also reversible and may prove to be useful in elucidating many ambiguous aspects of carcinogenesis and tumor recurrence in CRC patients. Furthermore, since epigenetic events can be reversed, we are able to regulate the process of chemical modification, including potential anti-neoplastic activity [7,10].

Among various epigenetic events, DNA methylation is a key component of epigenetics in mammalian genome that results in the addition of a methyl (CH_3_) group at the carbon-5 position of the cytosine ring. Two major methylation patterns are frequently observed in CRC: one is hypermethylation, which usually involves CpG islands, and the other is hypomethylation, which often involves repeated DNA sequences [6,8]. Although reports on hypermethylation in malignancies outnumber reports on hypomethylation, the latter seems to have become a topic of intense investigation in recent years [9,11]. In addition, methylation levels could be affected by sampling techniques, tumor locations, environmental factors, and the patient’s age [9,12–15].

Global DNA hypomethylation may lead to genetic instability. Its close relationship with a wide variety of malignancies, including CRC, has been reported in previous studies [6,7,9]. Retroelements, repeated sequences of long interspersed nuclear elements-1 (LINE-1), occur in almost 18% of the human genome [16]. These retroelements can cause deleterious effects in the host cell, but it is generally inhibited by suppressive mechanisms. However, retroelements may contribute to tumorigenesis in different settings. Global methylation can be measured indirectly by assessing the methylation status of LINE-1 [17]. Previous studies have demonstrated that the pyrosequencing assay for LINE-1 methylation is quantitative, effective, and reproducible [18–20].

Recently, many epigenetic studies have elucidated the relationship between LINE-1 methylation and cancers. It has been suggested that the degree of LINE-1 hypomethylation serves as an independent factor in determining unfavorable oncological outcomes in CRC patients [18,21,22]. Ogino et al. found that LINE-1 hypomethylation is an independent risk factor, indicating high cancer-specific mortality and overall mortality in colon cancer patients [21]. Sunami et al. also reported that there is a linear relationship between the progression of LINE-1 methylation and tumor progression [23]. Goel et al. reported that hereditary nonpolyposis colorectal cancer (HPNCC) was associated with lower degree of LINE-1 methylation [24]. In addition, some studies have found that early-onset CRC is associated with lower LINE-methylation and have highlighted its potential role as a prognostic biomarker in younger CRC patients [18,25]. However, many researchers have proposed LINE-1 as a promising marker for predicting CRC prognosis. Therefore, more patient-and treatment-related confounders should be controlled to generate more convincing conclusions, especially in light of modern strategies that highlight tailored treatments for different stages of CRCs.

Accordingly, in this study, we focused exclusively on the Stage III colon cancer population. We investigated the tumoral LINE-1 methylation status of stage III colon cancer patients after they were treated with radical resection and FOLFOX adjuvant chemotherapy. In this study, special attention was paid to their oncological outcomes, including postoperative recurrence and cancer-specific mortality. By controlling other potential prognostic factors, we sought to examine the prognostic impact of LINE-1 methylation in stage III colon cancer patients.

## Materials and Methods

### Ethics statement

The study was approved by the Institutional Review Board of Kaohsiung Medical University Hospital (KMUHIRB-20120043) in Kaohsiung, Taiwan. All participants in this study signed an informed-consent form.

### Study subjects

**Fig 1:**
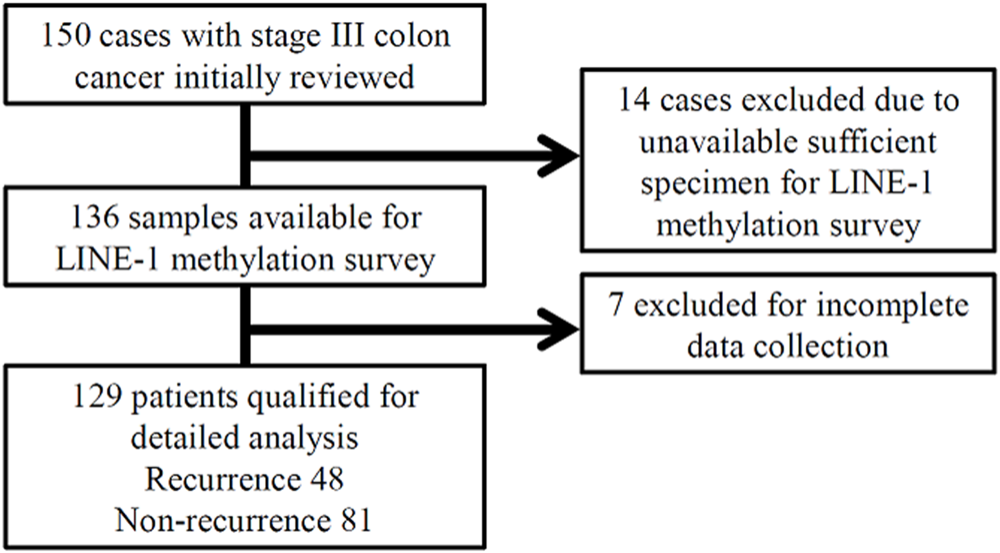
The algorithm used for study case selection.

We retrospectively reviewed the records of all colon cancer patients who were surgically treated at Kaohsiung Medical University Hospital between January 2008 and July 2012. A total of 150 stage III colon cancer patients who underwent radical resection and subsequent FOLFOX adjuvant chemotherapy were initially enrolled. Fourteen patients were excluded as optimal tissue specimens were unavailable. Among the remaining 136 patients, seven were excluded because the patients’ clinical data was incomplete. Thus, a total of 129 patients qualified for the final analysis (Fig 1). All patients received regular follow-ups at 2–3 month intervals until death or the end of June 2013.

### Postoperative adjuvant chemotherapy

All study participants received adjuvant FOLFOX-4 chemotherapy according to the center’s guidelines for cancer treatment. The following protocol was used for administering FOLFOX-4 chemotherapy: oxaliplatin 85 mg/m^2^ was infused within a two-hour period on Day 1; leucovorin 200 mg/m^2^ was infused concurrently with oxaliplatin within a two-hour period on Day 1, followed by a bolus of 5-FU 400 mg/m^2^; then a continuous infusion of 5-FU 600 mg/m^2^ was administered over 22 hours. This protocol was repeated every two weeks × 12 cycles.

### Clinical data collection

Patient characteristics, including age, sex, tumor location, and clinicopathological features, were obtained from electronic medical records and clinical charts. Clinicopathological features and the stages of primary tumors were defined using the criteria proposed by theAmerican Joint Commission on Cancer/Union for International Cancer Control (AJCC/UICC) [26]. Histology grades were classified as follows: well differentiated (WD), moderately differentiated (MD), or poorly differentiated (PD). As reported in a previous study, the tumor locations were categorized as proximal or distal to the splenic flexure [27]. Vascular invasion, perineural invasion, tumor invasion depth, and lymph node metastasis were factors particularly categorized before further comparison since each factor was considered as an independent risk factor [28–31].

### Genomic DNA isolation

These formalin-fixed, paraffin-embedded (FFRE) specimens obtained from 129 patients were reviewed to identify the representative sites of cancer. FFPE tissue blocks were microdissected to acquire 10 μm sections from each sample. Bisulfite treatment of tumoral DNA was then performed with an EpiTech bisulfite kit (Quiagen, Valencia, CA) according to the manufacturer’s protocol.

### Pyrosequencing for measuring LINE-1 methylation

In addition to the aforementioned sodium bisulfite DNA conversion process (EpiTech Bisulfite Fast FFPE Kits; Qiagen; No.59844), the pyrosequencing assays also comprised polymerase chain reaction (PCR) amplification (Pyromark PCR Kit; Qiagen; No.978703) and sequencing of the target sequence by synthesis assay (Pyromark Gold Q24 Reagents; Qiagen; No.970802). This assay amplifies a region of the LINE-1 element. The PCR conditions were 45 cycles of 95°C for 20 s, 50°C for 20 s, 72°C for 20 s, followed by 72°C for 5 min. The LINE-1 methylation results were obtained on a continuous scale of 1–100 using the pyrosequencing assay.

### Outcome measurements

The development of postoperative cancer recurrence and disease free survival (DFS) were used as major end-points to ensure that causal inferences about observed outcomes were mainly attributed to cancer. The development of a new local recurrence or distant metastatic lesions after surgery was considered as postoperative recurrences. While calculating DFS results, cases of postoperative mortality that occurred within 30 days were excluded. Individuals who died of causes other than CRC were also excluded from the outcome analysis. With regard to the treatment outcome for the FOLFOX regimen, DFS was defined as the time elapsed from the initiation of FOLFOX chemotherapy to recurrence.

### Statistical Analysis

The data were analyzed using the Statistical Package for the Social Sciences, Version 17.0 (SPSS. Inc., Chicago, IL). Student t-test, Pearson chi-square test, Fisher’s exact test, and the Kruskal-Wallis test were used to compare demographic and clinicopathological features between the groups according to their different outcome measurements. All the variables were further dichotomized for generating unadjusted odds ratio in the univariate analysis that was performed subsequently. Multiple logistic regression analysis was performed to identify variables that were significantly related to the likelihood of tumor recurrence or cancer-specific survival. Regression models were controlled for the effects of confounding variables. Results of the logistic regression analysis were reported as adjusted odds ratio (OR) with 95% confidence interval (CI). The comparative evaluation of long-term results was performed by analyzing the survival curves using Kaplan-Meier method. Curves for DFS were compared by Log-rank test. All tests of significance were two tailed, and *P*-values < 0.05 were considered statistically significant.

## Results

### Clinicopathological characteristics of the patient population

The mean age at surgery for these 129 patients was 61.6 years (range, 30–87 years), and 81 patients were male (62.8%). Clinicopathological features are detailed in Table 1. All the patients were treated with FOLFOX adjuvant chemotherapy. The median follow-up time was 24 months (IQR: 16.9–36). Tumor recurrence was reported by 48 patients, while 34 patients died during their follow-up period.

**Table 1:** Clinicopathological and demographic data of 129 stage III colon cancer patients.

Variable	N (%)
**Age, year (Mean, SD)**	61.6 (11.7)
**Sex, male**	81 (62.8)
**Tumor size≧5cm**	48 (37.2)
**Histology** ^**a**^	
** WD**	6 (4.7)
** MD**	105 (81.7)
** PD**	18 (14.0)
**T stage**	
** T1**	4 (3.1)
** T2**	12 (9.3)
** T3**	89 (69.0)
** T4**	24 (18.6)
**N stage**	
** N1**	87 (67.4)
** N2**	42 (32.6)
**Perineural invasion**	
** No**	86 (66.7)
** Yes**	43 (33.3)
**Vascular invasion**	
** No**	83 (64.3)
** Yes**	46 (35.7)
**Tumor location** ^**b**^	
** Right**	40 (31.0)
** Left**	89 (69.0)
**Oncological outcome**	
** Post-therapeutic recurrence**	48 (37.2)

^a^WD, well-differentiated; MD, moderately differentiated; PD, poorly differentiated.

^b^Tumor location is divided according to sites to the right or left of the splenic flexure.

### LINE-1 methylation analysis

**Fig 2:**
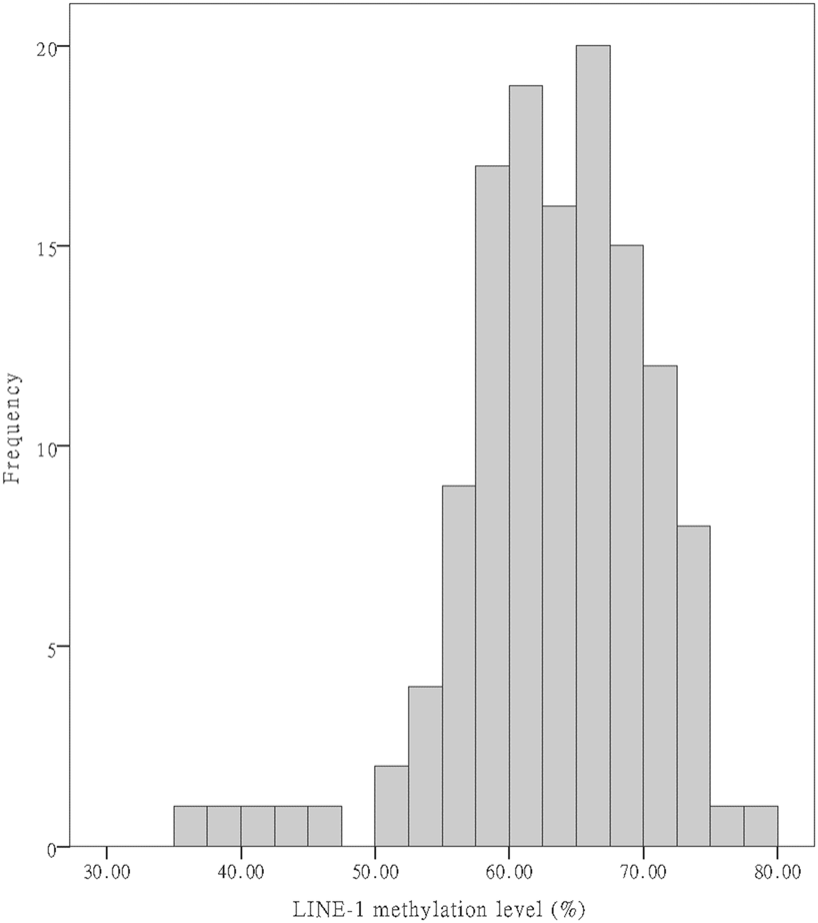
A histogram of patient LINE-1 methylation levels.

Although LINE-1 methylation levels in the 129 colonic tumors (Fig 2) were widely distributed (range, 37.4–77.9 on a 0–100 scale), they still showed normal distribution (Kolmogorov-Smirnov test; *P* > 0.05). The mean LINE-1 methylation level in ascending, transverse, descending, sigmoid colon and rectum, were 66.41±5.23%, 60.71±7.26%, 61.53±10.45%, 63.32±6.34%, 61.33±8.05%, respectively. The methylation level was significantly higher in ascending colon compared to other sites. (*p* = 0.042) The mean LINE-1 methylation level in the survival group was 63.8±6.6% versus 61.9±8.2% in the mortality group (*p* = 0.164). With respect to postoperative tumor recurrence, the mean LINE-1 methylation level was significantly lower in the recurrence group compared to the non-recurrence group (61.7±7.4% vs. 64.3±6.7%; *p* = 0.041). Because there is no universally accepted threshold to define whether the LINE methylation level is optimal or abnormal, we divided the levels of methylation into two groups using a receiver operating characteristic (ROC) analysis having balanced sensitivity and specificity. The cutoff value was 70.15% as the area under the curve (AUC) was 0.593.

### Univariate analysis of LINE-1 methylation level

We examined the LINE-1 methylation levels in the different groups categorized by the variables listed in Table 1. For a better comparison, age was dichotomized into two groups, with a cut-point value of 65 years. As shown in Table 2, in the univariate analysis incorporating nine variables, LINE-1 methylation levels were shown to be significantly different between the group aged 65 years and above and the group aged less than 65 years (64.6% versus 61.7%, respectively; *p* = 0.019).

**Table 2:** LINE-1 methylation status by demographic and clinicopathologic features.

Variable	N	LINE-1 methylation % (SD)	*p*-value
**Age <65 y/o**	73	64.6 (6.4)	0.019
** ≧65y/o**	56	61.7 (7.6)	
**Sex, male**	81	63.0 (6.6)	0.562
** Female**	48	63.8 (7.8)	
**Tumor size≧5cm**	48	62.2 (7.7)	0.156
** <5cm**	81	63.9 (6.6)	
**Histology** ^**a**^			
** WD**	6	64.4 (4.3)	0.481
** MD**	105	63.6 (6.6)	
** PD**	18	61.5 (9.8)	
**T stage**			
** T1**	4	68.2 (3.5)	0.195
** T2**	12	64.7 (6.8)	
** T3**	89	62.5 (6.3)	
** T4**	24	64.8 (9.6)	
**N stage**			
** N1**	87	63.8 (6.3)	0.238
** N2**	42	62.3 (8.5)	
**Perineural invasion**			
** No**	86	63 (7.1)	0.499
** Yes**	43	63.9 (6.9)	
**Vascular invasion**			
** No**	83	63.4 (6.6)	0.769
** Yes**	46	63.1(7.8)	
**Tumor location** ^**b**^			
** Right**	40	65 (6.3)	0.070
** Left**	89	62.6 (7.3)	

^a^ WD, well-differentiated; MD, moderately differentiated; PD, poorly differentiated.

^b^ Tumor location is divided according to sites to the right or left of the splenic flexure.

### Univariate and multivariate analysis of outcome measurements

Table 3 shows the results of the univariate and multivariate analysis with respect to the outcome measurements of post-therapeutic recurrence. The histology grades were divided into two groups, one incorporating well-differentiated (WD) and moderately differentiated (MD) specimens, and the other comprising poorly differentiated (PD) specimens. Due to a limited number of T1 cases, T-stages were dichotomized into “T1+T2” and “T3+T4” groupings. The LINE-1 methylation levels were dichotomized into “low” versus “high” groups according to a cutoff value of 70.15% generated by our previous ROC analysis. Thereafter, logistic regression was applied with forward selection. LINE-1 methylation appeared to be the only independent prognostic factor for the oncological outcome of post-therapeutic tumor recurrence (Adjusted OR = 14.1, *p* = 0.012).

**Table 3:** Univariate and multivariate analyses of relationships between postoperative recurrence and clinicopathological features of 129 stage III CRC patients.

Variable	Without Recurrence (N = 81) / N (%)	With Recurrence (N = 48) / N (%)	Univariate Analysis Unadjusted OR (95% CI)	*p*-value	Multivariate Analysis Adjusted OR (95%CI)	*p*-value
**Sex, male**	50 (61.7)	31 (64.6)	Reference		Reference	
** female**	31 (38.3)	17 (35.4)	0.884 (0.421–1.858)	0.746	0.973 (0.432–2.194)	0.948
**Age <65 years**	47 (58.0)	26 (54.2)	Reference		Reference	
** ≧65 years**	34 (42.0)	22 (45.8)	1.170 (0.570–2.401)	0.669	0.997 (0.456–2.183)	0.994
**Tumor size < 5cm**	52 (64.2)	29 (60.4)	Reference		Reference	
** ≧5cm**	29 (35.8)	19 (39.6)	1.175 (0.563–2.451)	0.668	1.016 (0.433–2.384)	0.970
**Histology** ^**a**^						
** WD +MD**	72 (64.9)	39 (81.3)	Reference		Reference	
** PD**	9 (11.1)	9 (18.8)	1.846 (0.677–5.032)	0.226	1.758 (0.552–5.596)	0.340
**T stage**						
** T1+T2**	12 (14.8)	4 (8.3)	Reference		Reference	
** T3+T4**	69 (85.2)	44 (91.7)	1.913 (0.580–6.308)	0.280	1.556 (0.411–5.895)	0.515
**N stage**						
** N1**	54 (66.7)	33 (63.8)	Reference		Reference	
** N2**	27 (33.3)	15 (31.3)	0.909 (0.423–1.955)	0.807	0.869 (0.362–2.086)	0.754
**Perineural invasion**						
** No**	50 (61.7)	36 (75.0)	Reference		Reference	
** Yes**	31 (72.1)	12 (25.0)	0.538 (0.243–1.187)	0.122	0.503 (0.205–1.234)	0.134
**Vascular invasion**						
** No**	52 (64.2)	31 (64.6)	Reference		Reference	
** Yes**	29 (35.8)	17 (35.4)	0.983 (0.466–2.073)	0.965	1.024 (0.451–2.321)	0.955
**Tumor location** ^**b**^						
** Right**	25 (30.9)	15 (31.3)	Reference		Reference	
** Left**	56 (69.1)	33 (63.8)	0.982 (0.454–2.124)	0.963	1.047 (0.437–2.512)	0.918
**Methylation level**						
** High**	18 (22.2)	1 (2.1)	Reference		Reference	
** Low**	63 (77.8)	47 (97.9)	13.429 (1.731–104.187)	0.002	14.107 (1.776–112.089)	0.012

Each risk factor was adjusted for all other risk factors listed in the table.

^a^WD, well-differentiated; MD, moderately differentiated; PD, poorly differentiated.

^b^Tumor location is divided according to sites to the right or left of the splenic flexure.

### Survival analysis

**Fig 3:**
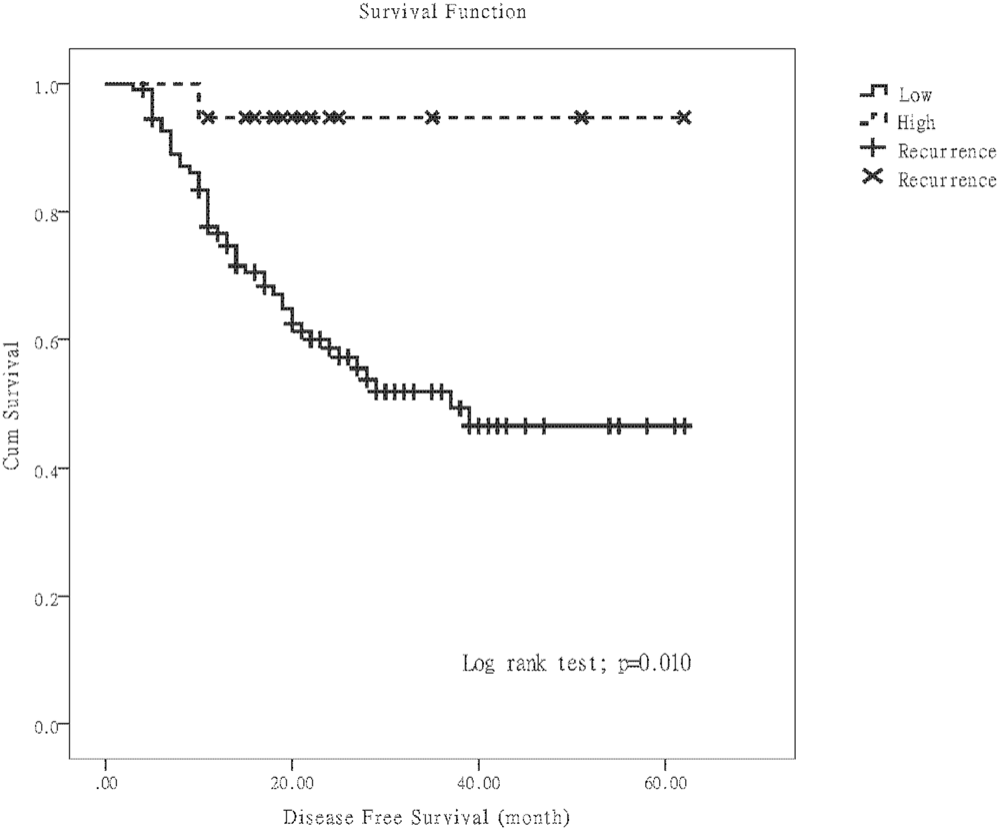
Kaplan-Meier disease-free survival curves. The curves depicting the effects on postoperative recurrence of LINE-1 methylation when divided into patients with “low” versus “high” methylation.

Follow-up data was available for all the 129 participants. The median survival period was 25 (IQR: 19–40) months for the patients who survived, 19.1 (IQR: 12.9–26.7) months for the patients who died, and 24 (IQR: 16.9–36) months for the overall cohort. To evaluate the prognostic implications of LINE-1 methylation, we focused on postoperative recurrence and DFS after taking into consideration the findings of multivariate analysis. The Kaplan-Meier method showed that more cases of postoperative recurrence and a shorter mean DFS were noted in the low LINE-1 methylation group (Fig 3, *p* = 0.010).

### Influence of LINE-1 methylation level on DFS in the patients with post-therapeutic recurrence

Based on the evidence regarding the shorter mean DFS in the low LINE-1 methylation group, we hypothesized that there is some association between LINE-1 methylation level and DFS in the recurrence group. In order to detect the threshold of DFS causing significant differences in LINE-1 methylation levels, we introduced four empiric thresholds of DFS (3, 6, 9, and 12 months) and dichotomized all recurrence patients into “early recurrence” (DFS shorter than or equal to the threshold) and “late recurrence2010043 (DFS longer than the threshold) groups. When the threshold of DFS is six months, the early recurrence group appeared to have a significantly lower mean LINE-1 methylation level (Table 4).

**Table 4:** The stratified analysis of LINE-1 methylation status in 48 stage III CRC patients with recurrence grouped by different thresholds of DFS.

Threshold of DFS (month)	Early Recurrence		Late Recurrence		*p*-value^b^
	N	Median (IQR) of LINE-1 methylation	N	Median (IQR) of LINE-1 methylation	
**3**	1	NA^a^	47	61.85 (8.79)	0.139
**6**	8	56.68 (15.75)	40	63.55 (7.57)	0.041
**9**	15	59.76 (11.76)	33	63.45 (7.27)	0.301
**12**	26	60.96 (10.56)	22	63.55 (7.38)	0.456

^a^NA, not available.

^b^tested by Wilcoxon Rank-Sum Test.

## Discussion

### Key findings of the present study

In this study, we demonstrated that LINE-1hypomethylation is associated with higher postoperative recurrence and a shorter DFS in stage III colon cancer patients, who have undergone radical surgery and subsequent FOLFOX chemotherapy. Previous studies have reported that the CRC patients’ tumor progression, survival, or recurrence may differ according to the level of LINE-1 methylation [21,23,32]. Nevertheless, as these studies included various population and different treatment procedures, there was obvious heterogeneity in their findings. We only assessed the prognostic implications of LINE-1 methylation levels on a homogenous population comprising stage III colon cancer patients, who had received radical resection and FOLFOX adjuvant chemotherapy. Our results confirmed the previously reported findings of Ahn et al. on the prognostic influence of LINE-1 hypomethylation on resected stage III CRC patients [33]. They emphasized that DNA hypomethyaltion was a feasible biomarker of recurrence in proximal but not in distal CRC cases. By including confounder adjustments, such as tumor location (proximal versus distal) in our study, we found that low LINE-1 methylation levels (below 70.15) still continued to be a robust risk factor having approximately eight times of postoperative recurrence risk.

### Relation between LINE-1 methylation and the subsite locations of tumors

The two-colon concept is conventionally used to indicate discrete molecular features via the dichotomous classification of proximal and distal colonic tumors. In previous studies, chromosomal instability (CIN) has been more commonly observed in distal CRCs [34]. On the other hand, microsatellite instability (MSI) and *BRAF* mutations are more common in proximal CRCs [35–37]. However, Yamauchi et al. recently found that the frequencies of high CpG island methylator phenotype (CIMP), MSI-high, and *BRAF* mutations gradually increase from the rectum to the ascending colon [38]. This finding profoundly challenged the conventional concept of the two-colon model that has been used for several decades. In our own data, although our comparison of LINE-1 methylation levels in right-sided and left-sided colon cancer patients indicated borderline statistical significance, we observed that the mean methylation level was significantly higher in ascending colon tumors compared to those in other sites. We recommend that the site-specific confounders should always be considered while conducting research studies on genetic or epigenetic alterations.

### The definition of hypermethylation or hypomethylation

Although a growing body of evidence supports the prognostic importance of LINE-1 methylation, a threshold defining whether the measured methylation level is hypermethylated or hypomethylated has yet to be established. Saito et al. observed the prognostic impact of LINE-1 methylation on non-small cell lung cancer keeping a high threshold of 90% [39]. Antelo et al. defined LINE-1 hypomethylation in young CRC patients using a cutoff value of 65%, and stated that those patients who had < 65% LINE-1 methylation had poorer overall survival [18]. In our study, we categorized the LINE-1methylation levels into high and low level groups based on previous researchers’ concepts [40,41]. In order to precisely identify an optimal cutoff value for LINE-1 methylation in patients reporting tumor recurrence after radical CRC surgery, we then conducted ROC analysis. For the continuous data obtained from these samples, the area under the curve (AUC) was found to be 0.593, and the 70.15% was considered as the cutoff value of methylation was with balanced sensitivity and specificity. With a higher cutoff value and poorer discriminative performance, our result was slightly different from that reported in the previous study by Baba et al [25]. In a heterogeneous population comprising CRCs of all stages, they achieved a better AUC of 0.62 by considering a cutoff value of 60%. Nevertheless, there is room for improving diagnostic performance as more large-scale studies must be based on stratified populations and different tumor locations.

### LINE-1 methylation variation in CRC with and without recurrence

Many researchers have enthusiastically continued exploring the mechanism by which LINE-1 methylation is associated with oncological outcomes. Hypomethylation of LINE-1 results in transcriptional activation of possible carcinogenesis [9,16]. An augmented expression of LINE-1 after hypomethylation may result in chromosomal instability (CIN), which has been termed as a characteristic phenotype in more invasive cancers having worse prognosis[13,16]. Furthermore, Hur et al. recently reported that there has been evidence to prove that LINE-1 hypomethylation serves as a crucial feature in CRC metastases, especially in hepatic metastasis [42]. This is in good agreement with the statistically significant difference between the LINE-1 methylation levels of recurrence and non-recurrence groups of our study (Table 2). Among the 48 colon cancer recurrences in our study cohort, there were 14 cases of hepatic metastases. However, the LINE-1 methylation levels of colon cancer patients with liver metastasis did not show a significant difference compared to those with other recurrences. In contrast with the perspectives of previous researchers, we have demonstrated a pivotal finding that LINE-1 hypomethylation has some influence on the early recurrence (DFS shorter than 6 months) in the cohort that eventually experienced recurrence. These results emphasize the crucial role of LINE-1 methylation in the development of colon cancer recurrence, especially early recurrence. Therefore, it is necessary to devise frequent and early surveillance strategies for guiding the post-operative follow-up of patients belonging to the hypomethylation group.

## Limitations

Several limitations must be considered while interpreting the findings of our study. First, some influential factors of global methylation could not be controlled. Because the study population was not prospectively recruited, certain environmental factors or patient-related information could not be recorded at surgery. For example, ionizing irradiation, smoking, and physical activity have been reported to cause variations in LINE-1 methylation [43–45]. To avoid introducing more recall biases, we didn’t try to control these potential confounders in our study. Furthermore, on the basis of this trial, it might be ethically unacceptable to conduct a study that randomizes patients with or without the administration of FOLFOX adjuvant chemotherapy. We suggested that methylation status could be used as a stratification factor. Second, the number of study participants is limited and the excluded population is still considerable. The excluded portion might possess some impact on the overall results if they were also enrolled. In addition, the follow-up duration could be extended to elucidate the prognostic impact on cancer-specific mortality. Despite these limitations, we were able to demonstrate that the clinicopathologic features were distinct and prognoses were different for the methylation subgroups formed from a homogenous Asian population.

## Conclusion

Our results indicate that despite being treated with radical surgery and FOLFOX adjuvant chemotherapy, stage III colon cancer patients with LINE-1 hypomethylation encountered early postoperative recurrences and shorter DFS.

## Supporting Information

S1 Dataset Details of the 129 study participants. (XLS)
